# Weller M. 25 Years of Ed Tech. Book series: Distance Education, AU Press, 2020. e-book, 211 pages, ISBN 9781771993067

**DOI:** 10.3325/cmj.2020.61.477

**Published:** 2020-10

Field of Medicine: Martin Weller’s 25 Years of Ed Tech offers a good opportunity for the reader to delve into a quarter-century history of educational technology (ed tech), which is perceived over the years as an emerging field. The book compiles the author’s texts published in his popular blog series. The focus is on higher education (HE), but even readers from other educational contexts, or who have not embraced online education, will find the book lively, informative, easy to read, and detailed but not overwhelming. Throughout the book, the reader will gain insight into how certain technologies have matured and become mainstream, while others have come and gone, either morphing into a different technology or failing because the world was not ready for them yet.

Audience: Educators, ed tech practitioners, and higher education administrators, as well as students.

Purpose: One of the author’s goals is to be a critical voice of ed tech, emphasizing its human and social role. Since ed tech field is remarkably poor at recording its own history, this book provides a contribution to ed tech history literature. Weller uses the history of ed tech to call for a shift from the often unquestioning advocacy of particular technologies to a more critical, theoretical understanding of their effect. He is focused on the potential learner, clearly detailing opportunities of ed tech development but also alerting about undesirable implications of technology adoption in educational settings.

Content: The book is divided into 25 chapters, each focusing on a technology, theory, or concepts that have marked a specific year since 1994. Through the chapters, it demonstrates a rich history of innovation in ed tech and its effective implementation across HE. It is clear that Weller's aim was not to write a comprehensive history of ed tech, but choose “one technology per year” based on his personal experiences and preferences. This choice is questionable as it potentially overlooks other technologies or technological movements. Despite all this, from bulletin board systems to blockchain, Weller has assigned technology to years based on his personal view of their significance or relevance in ed tech, rather than their time of invention.

The book starts with 1994, the year when the use of internet-related technology caused a fundamental shift in ed tech. In 1994, the Web was just about to enter mainstream, and the internet was gaining more popularity. The beginning was heralded by the Bulletin Board System (BBS), which raised the first real education system awareness of the possibilities of the internet. Since BBS had the potential to effectively remove the distance element, it could be considered as the precursor of the learning management system (LMS). As the author stated, the early applications of BBS sowed the seeds for social forums, social networks, and discussion groups.

In the second chapter, the reader is introduced to the world of the Web, known as Web 1.0. In 1995, the web browser was becoming reasonably commonplace, giving people the freedom to publish, communicate, and share. The Web enabled the spread of distance education, bringing the most significant socio-technological change since the invention of the printing press.

The following chapter discusses computer-mediated communication (CMC), which can be seen as the forerunner of LMS. Different types of CMC technologies introduced new communication formats through networked computers, allowing them to collaborate a distance. Weller describes the advantages and problems that emerged during the creation of the first online class using new graphical interfaces and functionalities.

Chapter 4 introduces the reader to constructivism, a learning theory based on the idea that learning is a process in which people actively construct their own knowledge out of experiences. Weller emphasizes the increase in its importance with the growth of the popularity of web-based learning. According to him, the constructivist approach has initiated a radical shift in the role of an educator in online learning environments from the “sage on the stage” to the “guide on the side.” Thus, constructivism offered a framework for the application of a number of specific pedagogical approaches, such as resource-based learning, problem-based learning, and communities of practice.

The technology that marked 1998 was Wikis, seen by the author as creating a fundamental shift in how we relate to the internet. By embodying the philosophy of the open web, Wikis showed great promise for educational implementation since they enabled students to work collaboratively on a document regardless of space or time. As Wikis' biggest success, the author recognizes Wikipedia, which had the potential to be a useful tool in HE but its use has waned partly because of emerging technologies such as Google Docs.

In 1999, it becomes obvious that e-learning was poised to become a part of mainstream HE. The development of the first fully online undergraduate course (Open University) necessitated the invention of a novel set of digital infrastructures and procedures. Its success ended the disputes about the potential of e-learning for distance education. In this chapter, Weller also elaborates the costs of e-learning, arguing against the belief that it would be cheaper than traditional education courses.

As an example of a technology that never really “took off,” Weller mentions learning objects (2000). He outlines their history and highlights the factors that hindered their success and potential wide-scale adoption. One of the factors was certainly the fact that they were and still are alien to many teachers, despite the compelling rationale for their existence.

By the turn of the millennium, e-learning has become established in education, and most universities started introducing some of its forms. This wide-scale adoption called for development of platforms that could be easily set up to deliver e-learning across an institution. Therefore, in Chapter 8, the author explains how the rise of e-learning has led to the development of various e-learning standards to describe content, assessment tools, and courses. The most significant standard was SCORM, which went on to become a standard for specifying the content of virtual learning environments.

One of the most successful education technologies, LMS, appeared in 2002. Chapter 9 focuses on the impact of the LMS on HE by offering a neat collection of the most popular tools. As an enterprise solution for e-learning, LMS provides a structured and safe environment to be implemented across HE institutions.

Chapter 10 is dedicated to the educational use of blogs, which entered mainstream by the mid-2000s. Blogging was seen as the start of what would become a networked academic identity. It is now obvious that nothing develops and anchors an online identity quite like a blog.

The mid-2000s also saw the rise of open educational resources (OER) movement, initiated by open-source software, which is nowadays growing into a global movement. The new open education movement is defined by its key element, a license that permits free use and repurposing. However, Weller also points out the drawbacks of OER, one of the greatest being their focus on content, often excluding the pedagogy and support structures.

In 2005, there appeared another valuable additional tool for educators, learners, and researchers – online video sharing, which played a tremendous role an in the democratization of broadcasts. Video promoted the emergence of a new pedagogical approach, “flipped learning concept.”

As a technology that marked 2006, Weller chose Web 2.0, since at that time the “Web 2.0” tag began to penetrate the educational usage. As an improved version of the first web, Web 2.0 has evolved into a medium where people share and upload user-generated content. Its usage was supported by social networks’ services, which inevitably led to the introduction of social media into education.

His pick of 2007 are virtual worlds. Although they had been around for some time, at that point they started gaining popularity among educators, and HE institutions began creating and delivering whole courses through Second Life and The Sims. Despite the fact that their educational potential did not gain as much traction with students as envisaged, Weller believes that virtual worlds as a learning tool could have a huge potential for a comeback.

Chapter 15 covers the use of e-portfolios within an educational context. As a place to store evidence of both formal and informal student learning, e-portfolios were intended to support lifelong learning and career development. However, despite the academic interest and investment, they were not adopted as a standard form of assessment.

Another revolutionary change in tech ed world took place in 2009, with the unexpected popularity of the use of Twitter and other social media as an education tool. Thanks to their ability to connect people all over the world, easily cross disciplines, and engage people in meaningful debates, Twitter’s social infrastructure and related social media profoundly changed the relationships among academics, students, and institutions. Although social media ultimately provided ed tech with a set of tools and possibilities, Weller warns that these benefits have not come without risks and issues.

In the following year, education saw the advent of connectivity. Educators began to explore the possibilities of education in a more networked (connected) model, which led to a new learning theory of connectivism. It is claimed to be “the first internet-native learning theory.” The key to connectivist approach is the belief that the best way to teach is to do so in open, digital environments, where the content is distributed anywhere online.

As a breakthrough of 2011, Weller chose personal learning environments (PLE), individual educational platforms that learners use to direct their own learning and pursue educational goals. Weller describes PLE as a combination of tools, people, and services that make up individualized resources and approach to learning. The difficulty in providing uniform support for learners, who were using different tools for data sharing, forced the educators to shift the focus from a personalized set of tools to a personalized set of resources. The PLE concept failed to realize its potential and was abandoned after its peak in 2011.

In 2012, there appeared a logical extension of the open education movement – massive open online course (MOOC). Combining several educational technologies such as OER, video, and Web 2.0 with connectivist approaches, MOOCs were seen as free and accessible tool with the potential to democratize education.

The year 2013 was dominated by open textbooks. According to Weller, the open textbook movement has proven to be one of the most amenable to the open approach, with considerable cost reduction and pedagogical benefits.

A year later, the focus was on learning analytics, which refers to the collection and analysis of data about learners and their environments for the purpose of understanding and improving their learning outcomes. The author argues that the interest in analytics was driven by the need to adjust the course material or content based on student behavior. He also uses this topic to issue a warning to universities about careful and ethical handling of the collected data to avoid mistakes such as irresponsible data distribution.

A good example of how ed tech evolves with coalescence of several other technologies are digital badges. They entered education in 2015 as a tool to validate the accomplishment, skill, or competences earned in online learning environments. Like many other ed tech developments, digital badges sparked an initial flurry of interest from devotees, but later on this interest turned into a long-term acceptance.

The year 2016 was marked by the rise of artificial intelligence (AI), which was not a new technology in ed tech. AI refers to the simulation of human intelligence by smart machines programmed to respond like humans. The initial enthusiasm over the technology in 1980s was diminished by the limitations of computer power and capabilities to process the complexity of information. However, the field was reignited by faster computers, algorithmic improvements, and access to large amounts of data. Despite the successful implementation of AI in education, Weller argues for caution regarding the ethical implications and moral questions arising from its use.

In 2017, the focus switched to blockchain, the application that can be seen as a digital tool to store, track, and use students’ records of academic achievement and digital credentials. Blockchain in education brings together different technologies, such as e-portfolios, digital badges, MOOC, OER, PLEs, and personalized learning. Weller is skeptical about this technology, primarily because blockchain adoption has been seen as an end goal in itself, rather than as an appropriate solution to a specific problem.

In the last chapter, covering 2018, the author focuses on a trend rather than technology, and explores the dystopian view of educational technology and its implications. Weller is conscious of the critiques of the role of technology in society and education. He knows that a challenge in this field is to directly engage educators in the development of ed tech since much of the innovations have been developed by technologists from other industries. Technologists often avoid engaging educators in the design, development, and deployment of technological innovations, but unfamiliarity with ed tech history makes them repeat the same mistakes over and over again. According to Weller, universities can counteract this problem by bringing critical, research-based approaches to ed tech.

Highlights: The book ends with a reflective chapter, in which the author suggests that building on past successes and learning from failure could be a better approach than constant rediscovery of ed tech. Weller sees the future as a relationship between people, technology, and knowledge, which leads to learning. The ed tech developments should be better balanced with conceptual frameworks, pedagogies, and social movements. It is obvious that a considerable shift in higher education teaching has taken place, driven by technology adoption. Yet, at the same time, nothing much has changed. The influence of technology on education Weller best explains by: “Everything changes while simultaneously remaining the same”.

